# End-to-End Platform for Human Pluripotent Stem Cell Manufacturing

**DOI:** 10.3390/ijms21010089

**Published:** 2019-12-21

**Authors:** Puspa R. Pandey, Amarel Tomney, Marites T. Woon, Nicholas Uth, Farjad Shafighi, Igor Ngabo, Haritha Vallabhaneni, Yonatan Levinson, Eytan Abraham, Inbar Friedrich Ben-Nun

**Affiliations:** Cell and Gene Therapy Research and Development, Lonza Inc., Rockville, MD 20850, USA; puspa.pandey@lonza.com (P.R.P.); amarel.tomney@gmail.com (A.T.); marites.woon@lonza.com (M.T.W.); nicholas.uth@lonza.com (N.U.); Farjad.shafighi@lonza.com (F.S.); Igor.ngabo@lonza.com (I.N.); hvallab1@hotmail.com (H.V.); yonatan.levinson@lonza.com (Y.L.); eytan.abraham@lonza.com (E.A.)

**Keywords:** human pluripotent stem cells, bioreactors, automation, allogeneic cell therapy, differentiation, expansion, microcarriers, kSep

## Abstract

Industrialization of stem-cell based therapies requires innovative solutions to close the gap between research and commercialization. Scalable cell production platforms are needed to reliably deliver the cell quantities needed during the various stages of development and commercial supply. Human pluripotent stem cells (hPSCs) are a key source material for generating therapeutic cell types. We have developed a closed, automated and scalable stirred tank bioreactor platform, capable of sustaining high fold expansion of hPSCs. Such a platform could facilitate the in-process monitoring and integration of online monitoring systems, leading to significantly reduced labor requirements and contamination risk. hPSCs are expanded in a controlled bioreactor using perfused xeno-free media. Cell harvest and concentration are performed in closed steps. The hPSCs can be cryopreserved to generate a bank of cells, or further processed as needed. Cryopreserved cells can be thawed into a two-dimensional (2D) tissue culture platform or a three-dimensional (3D) bioreactor to initiate a new expansion phase, or be differentiated to the clinically relevant cell type. The expanded hPSCs express hPSC-specific markers, have a normal karyotype and the ability to differentiate to the cells of the three germ layers. This end-to-end platform allows a large scale expansion of high quality hPSCs that can support the required cell demand for various clinical indications.

## 1. Introduction

Successful generation of human-induced pluripotent stem cells (hiPSCs) by somatic cell reprogramming has opened new avenues in regenerative medicine, disease modeling and drug development [1,2]. Capable of self-renewal and pluripotency, hiPSCs derived from patients of both normal and aberrant phenotypes provide a theoretically limitless supply of clinically relevant iPSC-derived cells without ethical limitations and immune-rejection [3,4]. For instance, given the heart’s limited-to-no regenerative capacity, new cardiomyocytes can be derived from hiPSCs by modulating developmental cues critical in embryonic development in vivo [5,6]. Essential to the successful differentiation of iPSCs to a specific cell lineage, however, includes careful consideration of the microenvironment and method with which iPSCs are maintained. While a wealth of information has been gained through the use of traditional, two dimensional (2D) culture, this system fails to generate the number of cells required in many therapies in a cost-effective manner, and does not fully recapitulate in vivo conditions. To replace the number of cells lost during a myocardial infarction, for example, approximately 1 × 10^9^ cells are required per patient dose [7].

Given that 2D-based cell culture platforms are nonscalable with minimal capacity for expansion, achieving high cell densities in a 2D system would involve costly arrangements including extensive manual effort, laboratory space and personnel. These platforms also often do not possess adequate systems to control or monitor parameters, such as the production of key metabolites by hiPSCs in culture. Moreover, iPSC-derived cardiomyocytes remain phenotypically immature [8], despite a number of studies demonstrating enhanced maturation through the modulation of existing methodologies [9,10,11,12,13]. 

Recent innovations in suspension culture systems provide robust, controlled and scalable platforms beyond conventional 2D approaches, which can be translated to current Good Manufacturing Practice (cGMP) compliant processes [14,15,16]. A number of studies have demonstrated the feasibility of hPSC expansion in suspension cultures using aggregate [14,16,17] and microcarrier (MC)-based [18,19,20] three dimensional (3D) culture systems. Aggregate-based 3D culture provides a more physiologically relevant microenvironment, but has been shown not only to require the small molecule, Y27632, for the survival of hPSCs [15], but also sequential passaging to achieve high fold expansion [21]. Not without its own advantages, microcarrier-based culture systems facilitate a larger surface area to volume ratio for scalability, provide large surface area for adhesion and growth during expansion, flexibility in using defined extracellular matrices, and allow the maintenance of homogenous culture conditions [15,19,20].

**Figure 1:**
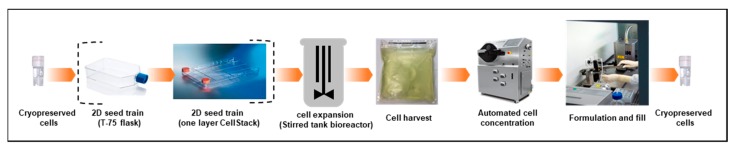
Schematic representation of the end-to-end microcarrier-based human pluripotent stem cells (hPSCs) expansion platform. This platform includes the following attributes: perfused, xeno-free, proprietary media, plastic microcarriers (MCs) coated with proprietary matrix, high fold expansion without cell passaging, and a closed concentration step. The two-dimensional (2D) seed train could be avoided by using cryopreserved cells as the inoculum.

Considering the need for a cGMP compliant, commercially viable, scalable process to generate large numbers of high quality hPSCs, we present an end-to end platform for hPSC expansion (Figure 1). The expansion is microcarrier-based, and uses xeno-free culture conditions. Cells are expanded in a closed, automated and controlled single-use stirred tank bioreactor. Following a closed step of harvest and separation from the MCs, the cells are concentrated using a closed, automated centrifugation system and are further cryopreserved. The starting material, cryopreserved hPSCs, are either thawed into 2D culture prior to inoculation, or thawed directly into a bioreactor. A high expansion fold of >50 is achieved using this platform within 10–14 days of culture. Expanded hPSCs demonstrate a high quality of self-renewal and pluripotency, and are capable of differentiation to all three germ layers.

## 2. Results

### 2.1. L7™ TFO2 Human Pluripotent Stem Cells (hPSC) Media Supports hPSC Growth and Expansion in Suspension on L7™ hPSC Matrix-Coated Microcarriers

The L7™ TFO2 hPSC medium supports human iPSC generation from somatic cells, such as fibroblasts and PBMNCs, and the maintenance of various hESC and hiPSC lines in conventional 2D cell culture platforms, which utilize cell culture vessels coated with this L7^TM^ hPSC Matrix to support cell attachment. To assess the ability of L7™ TFO2 hPSC media to support the growth of hPSCs in suspension, 2D-cultured RTiPSC3B and RTiPSC4i cells were harvested as cell clumps and inoculated in L7™ TFO2 hPSC medium at a cell density of 0.2 × 10^6^ cells/mL, with MCs 90–150 µM in diameter coated with the L7^TM^ hPSC Matrix. 

**Figure 2:**
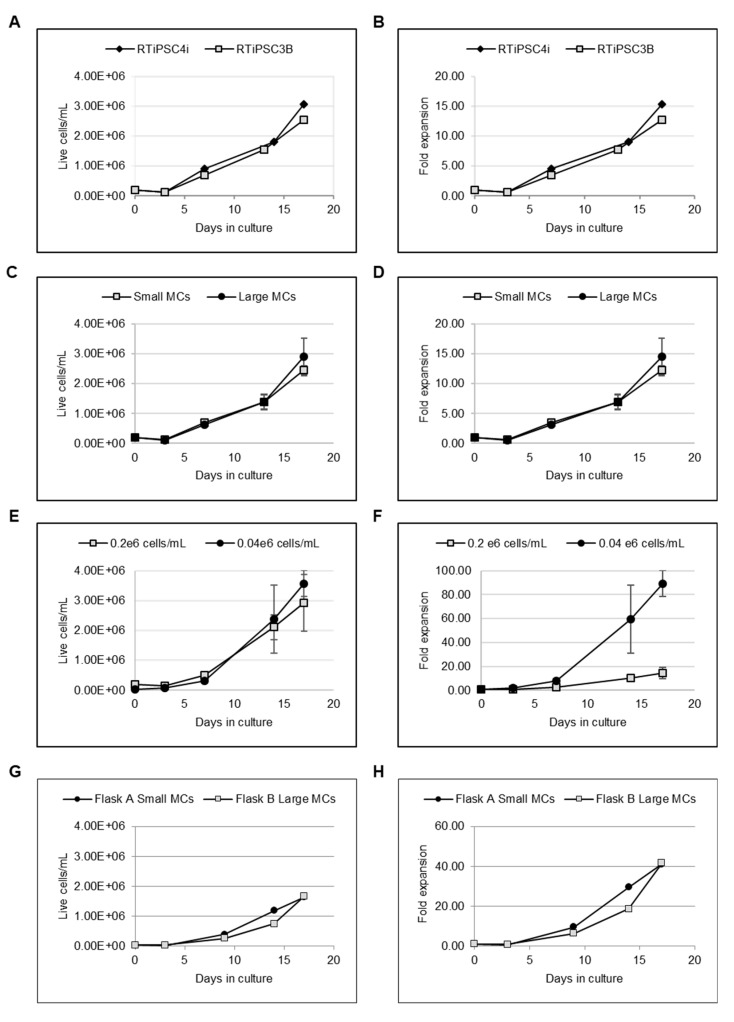

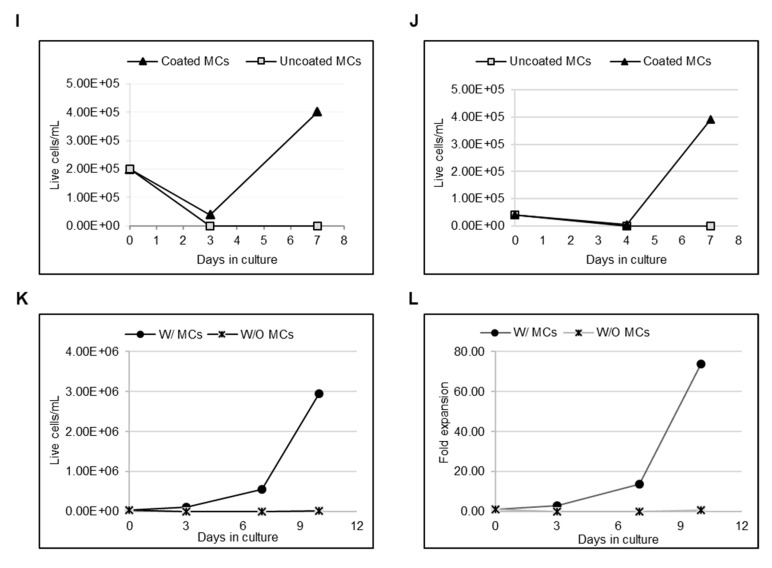
L7™ TFO2 hPSC xeno-free media supports the microcarrier (MC)-based expansion of RTiPSC3B and RTiPSC4i human-induced pluripotent stem cells (hiPSCs) leading to >2 × 10^6^ cells/mL (**A**) and >10 fold expansion (**B**) in 17 days. Small- and large-sized MCs support the cell growth (**C**) and expansion (**D**) of RTiPSC3B in L7™ TFO2 hPSC xeno-free media. Inoculating less cells/mL does not compromise cell yield, but leads to rapid cell growth and higher cell expansion of RTiPSC4i (**E**,**F**) and RTiPSC3B (**G**,**H**), which were cultured on small- and large-sized MCs. Coating MCs with the L7™ hPSC Matrix is essential for the attachment and expansion of hiPSCs in L7™ TFO2 hPSC medium. RTiPSC3B inoculated at 0.2 × 10^6^ cells/mL with small-sized microcarriers (**I**) and LiPSC18R inoculated at 0.04 × 10^6^ cells/mL with large-sized microcarriers (**J**). RTiPSC4i inoculated at 0.04 × 10^6^ cells/mL with large-sized coated and uncoated microcarriers. Cell growth (**K**) and fold expansion (**L**) are shown.

As indicated in Figure 2A,B, an initial decrease in cell number was observed during the first days in suspension followed by an increase in cell number, which reached >2 × 10^6^ cells/mL on day 17 (>10-fold expansion). The results indicate that L7™ TFO2 media supports MC-based expansion of hPSCs in suspension. Likewise, our findings demonstrate that 10-fold expansion could be achieved in a continuous suspension culture over 17 days without the need for cell passaging.

### 2.2. Large-Sized Microcarriers Support Expansion of hPSCs Comparably to Small-Sized Microcarriers

Given that MCs of larger diameters do not require custom manufacturing and have better availability, we tested whether the diameter of MCs impacts cell attachment and growth. Spinner flasks were inoculated with 0.2 × 10^6^ cells/mL of RTiPSC3B in the presence of 90–150 µM (small) or 125–212 µM (large)-sized MCs coated with L7™ hPSC Matrix. Similar cell growth and expansion over 17 days was observed when using small and large-sized MCs. In both conditions, cells reached >2 × 10^6^ cells/mL and >10-fold expansion on a similar day during culture (Figure 2C,D, respectively). These results validate the use of larger-sized MCs for cell expansion.

### 2.3. Lower Inoculation Cell Density Does Not Compromise Cell Yield, But Leads to a Higher Fold Expansion

Conventional expansion of hPSCs in 3D culture primarily relies on 2D-cultured cell inoculum [17,20,22]. To generate high cell numbers in suspension systems such as a 3 L bioreactor with an inoculum cell density of 0.2 × 10^6^ cells/mL, would require 600 × 10^6^ cells at inoculation. Considering the extensive, manual and open 2D cell culture effort that will be required to meet this number, we wanted to determine whether the inoculum of a lower cell density would result in cell expansion, and if so, how long it would take to achieve a cell yield of >2 × 10^6^ cells/mL.

Spinner flasks were inoculated with RTiPSC4i cell clumps at two different cell densities: 0.2 × 10^6^ cells/mL (Condition A) and 0.04 × 10^6^ cells/mL (Condition B). The cells were cultured in suspension for 17 days, and cell counts were determined at various time points during the expansion period. Our results show that comparable cell densities were achieved in both Conditions A and B. The spinner flasks inoculated with a higher cell density (Condition A) resulted in 3 × 10^6^ cells/mL, while inoculating with a lower cell density (Condition B) resulted in 2.5 × 10^6^ cells/mL on day 17 (Figure 2E). Comparison of expansion fold results (Figure 2F) demonstrates that Condition A resulted in ~15-fold expansion by day 17, which is comparable to the expansion fold described above (Figure 2B). However, Condition B, which used five times fewer hiPSCs at inoculation, resulted in 90-fold expansion on day 17 (Figure 2F). This fold expansion is ~6 times higher than that obtained in Condition A. To confirm these findings with a different hiPSC line, we inoculated RTiPSC3B cells at a cell density of 0.04 × 10^6^ cells/mL, with either small- or large-sized coated MCs. Figure 2G,H demonstrate cell growth and fold expansion over 16 days. Cell yield reached >1.6 × 10^6^ cells/mL with an expansion fold of >40 on either small- or large-sized MCs. Our findings show that hiPSCs inoculated at lower cell densities are capable of achieving higher-fold expansion, easing the burden of largescale expansion in 2D cell culture platforms prior to inoculation in 3D suspension culture.

### 2.4. L7™ hPSC Matrix Coating Enables hPSC Attachment to Plastic Microcarriers

A short-term experiment of seven days was conducted with RTiPSC3B and LiPSC18R cells to determine whether any coating of microcarriers with L7™ hPSC Matrix is indeed needed to support hPSC attachment to the microcarriers. Cells were harvested from 2D cell culture using L7™ Passaging solution and seeded as cell clumps in spinner flasks at a density of 0.2 × 10^6^ cells/mL (RTiPSC3B) and 0.04 × 10^6^ cells/mL (LiPSC18R). Both flasks for each cell line contained microcarriers, where in one flask the microcarriers were coated with the L7™ hPSC Matrix and in the other, the microcarriers were not coated. While cell viability on day 0 (inoculation day) was determined to be 85%, cell counts performed on day 3 (RTiPSC3B) and day 4 (LiPSC18R) revealed a decrease in cell number in the presence of coated and uncoated microcarriers (Figure 2I–J, respectively). This observation matches previous results for reduced cell density in the first days in suspension (Figure 2A,C). On day 7, however, RTiPSC3B cells that were seeded with L7™ hPSC Matrix-coated MCs showed both high viability (90%) and 2-fold expansion, while LiPSC18Rexpansion resulted in 10-fold expansion (Figure 2I–J, respectively). Cells that were seeded with uncoated MCs failed to show growth and expansion (Figure 2I–J). These results demonstrate that coating the MCs with L7™ hPSC Matrix is required for cell expansion. To rule out the possibility that the cells expanded without MCs, RTiPSC4i cells were inoculated in spinner flasks with and without large-sized MCs coated with this L7™ hPSC Matrix. In the flask that had MCs, cells reached 70-fold expansion in 10 days, while no expansion was detected in the flask that did not have MCs (Figure 2K,L). This data shows that MCs are needed to support cell expansion.

### 2.5. High Fold Expansion of hPSCs in a Stirred Tank Bioreactor

**Figure 3:**
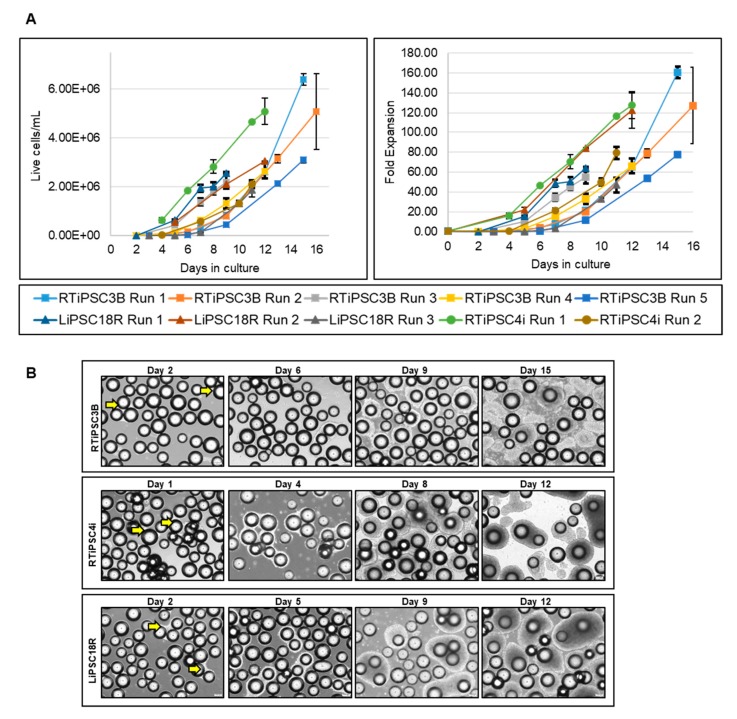
Expansion of hiPSCs in 3 L bioreactor suspension cultures. (**A**) Consolidated cell density (left panel) and fold expansion (right panel) results from ten bioreactor runs using 2D-cultured RTiPSC3B, RTiPSC4i and LiPSC18R cell inoculum; (**B**) Representative images of cell-MC clusters on various days of the bioreactor run. Images are shown for RTiPSC3B, RTiPSC4i and LiPSC18R cell lines. Arrows indicate cells growing on MCs on the indicated days. Magnification, 100×; Scale bar: 200 µm.

Our spinner flask experiments demonstrated that hiPSCs which were expanded in L7™ TFO2 media using L7™ hPSC Matrix-coated MCs resulted in high-fold expansion. To demonstrate scalability, we tested our 3D expansion system in 1 L and 3 L stirred tank bioreactors. Ten 3 L bioreactor runs were performed using three different hiPSC lines harvested from 2D culture as cell clumps. Cells were inoculated at a cell density range of 0.02–0.04 × 10^6^ cells/mL with L7™ hPSC Matrix-coated MCs (125–212 µM) and maintained in L7™ TFO2 media, perfused at 1 vessel volume per day (VVD). Cell counts performed on single cells released from the MCs revealed an expansion of 5- to 20-fold in the first 5–9 days, and an additional >10 fold expansion onward, resulting in >2 × 10^6^ cells/mL (Figure 3A). An average 93-fold expansion within 9–16 days of culture across all three hiPSC cell lines was achieved in these culture conditions. Notably, although RTiPSC4i Run 2 used only 0.027 × 10^6^ cells/mL at inoculation, approximately 80-fold expansion was still achieved in 11 days. This result supports our findings in spinner flasks’ experiments, demonstrating that lower inoculum cell density does not compromise cell yield, supporting platform robustness. Cell viability along the various days of the bioreactor’s run was determined to be high (>85%). Images of cell-MC samples taken from the bioreactor at various time points show cell expansion over time (Figure 3B).

**Figure 4:**
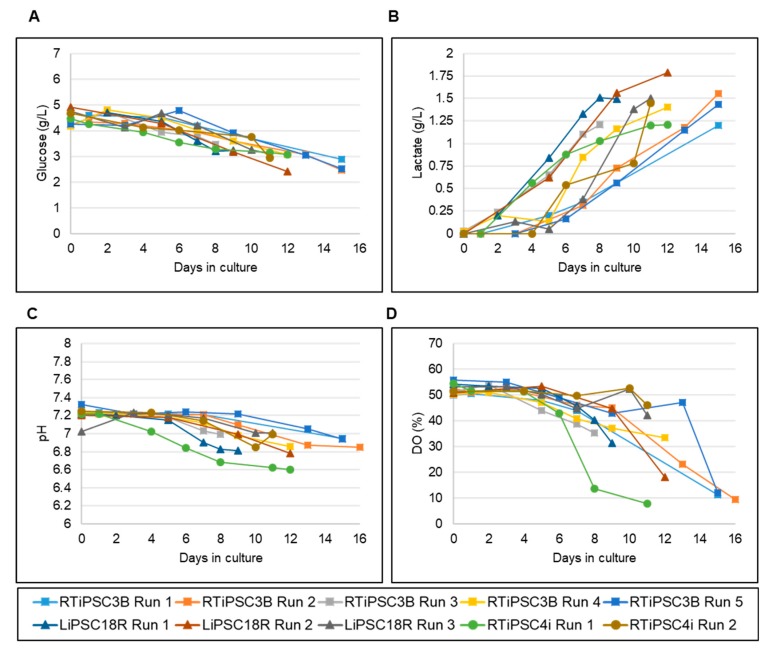
Monitoring of nutrient and metabolite concentrations and process parameters in 3 L bioreactor suspension cultures of hiPSCs. Offline monitoring of glucose consumption (**A**) and lactate production (**B**). Online monitoring of pH (**C**) and dissolved oxygen (DO) (**D**).

Since nutrient consumption and production of metabolites during expansion can inform us of the quality of expanded hiPSCs [17], we periodically measured key nutrients, such as glucose, and metabolites like lactate, during the runs. Figure 4A shows a decrease in glucose levels, corresponding to increased glucose consumption as the cells expand in culture. Glucose concentrations did not drop below 2.4 g/L, even when the cell density in the bioreactor reached 3.05 × 10^6^ cells/mL (LiPSC18R Run 2). Conversely, lactate production increased with a maximum concentration of 1.79 g/L seen (Figure 4B, LiPSC18R Run 2). For the other cells lines, lactate levels did not exceed 1.6 g/L even for high cell densities of 5.6 × 10^6^ cells/mL (RTiPSC3B) or 5.1 × 10^6^ cells/mL (RTiPSC4i). Additionally, pH and dissolved oxygen (DO) were closely monitored in real time using the TruBio DV Software from Finesse Solutions. Similarly to the nutrient levels, the pH levels were affected by cell expansion. While the set point was 7.2, as cells expanded, pH levels dropped to ~6.8.

Dissolved oxygen was set to 50% and maintained for the first few days of the run. As the cell expanded, however, DO levels dropped and the Finesse controller could not maintain the set target (Figure 4B). These DO levels dropped to 30% for cell densities close to 2 × 10^6^ cells/mL. For higher cell densities of >4 × 10^6^ cells/mL, DO levels were below 10%.

### 2.6. Quality Assessment of hPSCs Post-Harvest after Expansion in Stirred Tank Bioreactors

**Figure 5:**
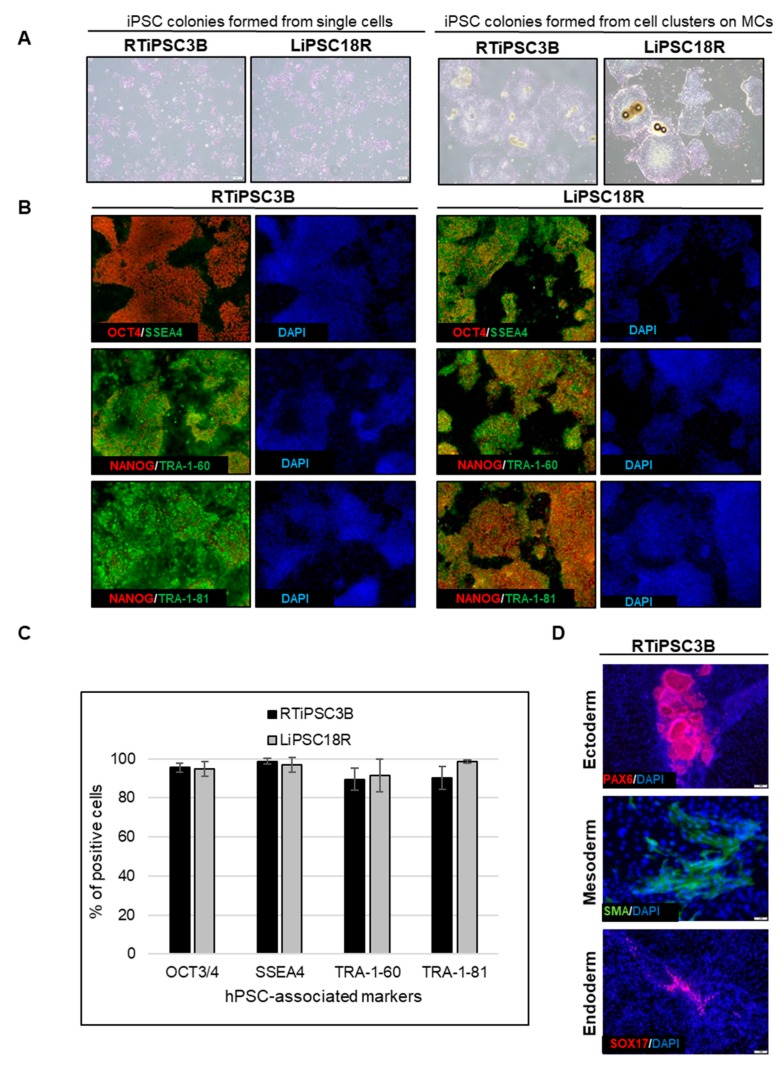

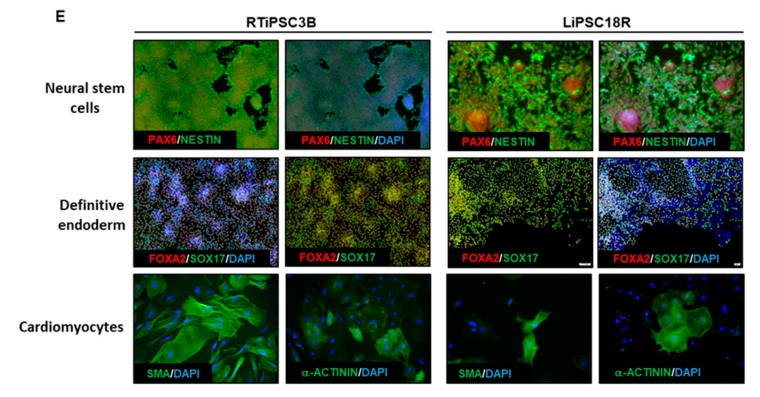
Human iPSCs expanded in a bioreactor have typical hiPSC morphology and express hPSC-associated markers post-harvest. (**A**) Phase contrast images of iPSCs expanded in a bioreactor plated either as single cells after cell release from MCs (left panel), or as clusters of cells on MCs (right panel). Magnification, 100×; Scale bar: 100 µm; (**B**) Immunofluorescence staining of OCT3/4, NANOG, SSEA-4, TRA-1-60 and TRA-1-81 of iPSCs expanded in a bioreactor and plated onto 2D cell culture plates. Representative images from two different cell lines are shown. Magnification, 100×; (**C**) Quantitative analysis of hPSC-associated markers OCT3/4, SSEA4, TRA-1-60 and TRA-1-81 by flow cytometry of RTiPSC3B and LiPSC18R cells expanded in a bioreactor. Data obtained from either three independent runs (RTiPSC3B) or two independent runs (LiPSC18R). Data plotted are mean ± standard deviation (SD); (**D**) Pluripotency of RTiPSC3B cells expanded in a bioreactor demonstrated by embryoid body (EB) formation followed by immunofluorescence staining for germ layer-specific markers. Magnification, 100× (ectoderm, endoderm), 200× (mesoderm). (**E**) Representative immunofluorescence staining for lineage-specific markers RTiPSC3B and LiPSC18R cell lines. Magnification, 100× (Neural stem cells, definitive endoderm), 200× (cardiomyocytes)

Cell harvest from the bioreactor was performed in a closed manner, inside the bioreactor. Medium was pumped out, and F3 non-enzymatic passaging solution was pumped in to release the cells from the microcarriers. As a result of this F3 passaging solution treatment, cells were released from the microcarriers as single cells. The resulting solution of single cells and microcarriers in this F3 passaging solution were then transferred in a closed manner through a separation bag to separate the cells from the microcarriers. The cells passed through the filter bag directly into a collection bag containing L7™ TFO2 hPSC media. In order to assess the quality of expanded hiPSCs post-harvest from a bioreactor, we characterized the cells for morphology, hPSC-associated markers, to validate their karyotype, and determine their pluripotency. To assess morphology, hiPSCs on MCs or MC-released hiPSCs were plated onto 2D cell culture plates coated with L7™ hPSC Matrix. Figure 5A (left panel) shows that RTiPSC3B and LiPSC18R cells, cultured in 2D culture plates after harvest from a 3 L bioreactor as single cells, attached well and grew as a monolayer with small colonies observed on day 3. Meanwhile, cell-MC clusters plated directly onto 2D culture plates without releasing the cells as single cells, exhibited typical hPSC colony morphology, including defined edges [23] and tightly packed cells with large nuclei and scant cytoplasm (Figure 5B, right panel) [1].

Moreover, immunofluorescence staining of both cell lines shows qualitative expression of hPSC-associated markers (Figure 5B), while flow cytometry results further validated that >85% of the iPSCs expanded in the bioreactor-expressed, hPSC-associated markers post-harvest (Figure 5C). Karyotype analysis showed no genome abnormality in cells harvested from a bioreactor and cultured for one or more passages in 2D (Table 1).

The potential of the expanded hiPSCs to differentiate into cells of the three germ layers was assessed either by embryoid body (EB) formation or by directed differentiation. Figure 5D shows immunofluorescence staining images of plated EBs formed from RTiPSC3B cells expanded in a 3 L bioreactor. Positive detection of germ layer-specific markers demonstrates that cells which expanded in a bioreactor retain their potential to give rise to cells of the three germ layers post-harvest. Directed differentiation of hiPSCs to definitive endoderm (DE, endoderm germ layer), neural stem cells (NSCs, ectoderm germ layer) and cardiomyocytes (CMs, mesoderm germ layer) was performed on RTiPSC3B and LiPSC18R cells after harvest from 3 L bioreactors. Immunostaining for germ-layer-specific markers confirmed that the expanded cells retained pluripotency and the capability to be directly differentiated to DE, NCSs and CMs (Figure 5E). As described in our Materials and Methods section, immunostaining for CM-specific markers was performed after observing spontaneous contraction.

### 2.7. Cell Concentration by kSep: Flow Rate Optimization

As the manufacture of cell therapies advances towards larger scale cultures in bioreactors, more cells will be used for inoculation, and more cells will need to be processed after harvesting. Common treatment of iPSCs after a bioreactor harvest is to have an operator wash out the culture media, concentrate the cells via benchtop centrifugation, and then re-suspend in cryoprotectant. Moving towards larger scale GMP production, these open steps are a critical contamination risk to the product, and ultimately to the patient who would receive it, and are difficult to scale. One solution is to make use of continuous centrifugation devices like the kSep400 system.

Optimization of the flow rate during fluidized bed formation was defined as (1) minimizing the time needed to establish the fluidized bed, (2) maximizing cell recovery and (3) maintaining cell viability and proliferation capabilities. The establishment of the fluidized bed is achieved when the majority of cells coming into the kSep chamber are retained, and thus the percent of escaping cells reaches a minimum. Quantitatively, this can be defined as the escape percent dropping below 10%, meaning the fluidized bed captures 90% or more of the incoming cells.

The kSep 400.50 is a 1/3.5 scale-down version of the kSep400, which is used for process optimization; because fewer cells are needed to run in the kSep 400.50, more experimental conditions can be tested with fewer cells. To optimize the formation of the fluidized bed, three flow rates (25, 30 and 35 mL/min) were tested in increasing order. For the kSep 400.50, this range of flow rates is where typically-sized cells will equilibriate in the kSep chambers, and 30 mL/min is where Sartorius recommends starting the optimization experiments.

**Figure 6:**
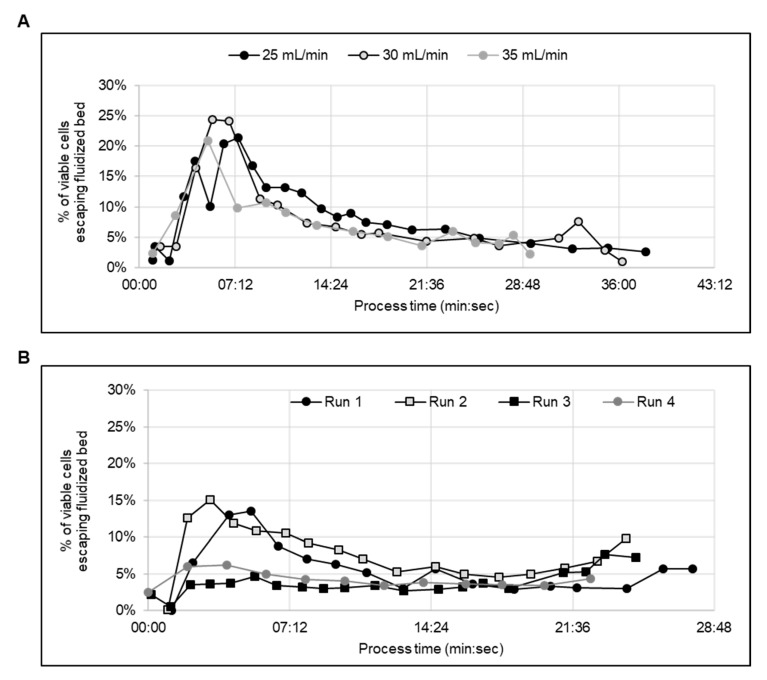
Concentrating hiPSCs by automated centrifugation. (**A**) Comparison of the viable cells which escape the kSep 400.50 chamber during the fluidized bed formation step. (**B**) Comparison of viable cells escaping the fluidized bed per kSep400 run versus the process time.

The 30 mL/min and 35 mL/min flow rates established the fluidized bed in 10–11 min, and the 25 mL/min flow rate established the bed after 13–14 min (Figure 6A). The cells from the 30 and 35 mL/min tests were taken for cell counts and culturing. The cell counts revealed that the cell viability was not negatively affected by the concentration process, and that both protocols had recoveries ≥80% (Table 2). The flow rate of 35 mL/min was selected for the concentration of iPSCs in the kSep 400.50. This flow rate was scaled up to 120 mL/min when moving into the kSep400. At this flow rate, a 50 L volume of cell suspension could be processed in under two hours (105 min, or 110 min, including priming and harvesting).

### 2.8. Concentrating Cells Post Expansion in a Stir Tank Bioreactor

After establishing a possible flow rate (120 mL/min) for both the fluidized bed setting and the concentration steps, five bioreactor harvests were concentrated using the kSep400. For four of these runs, the waste stream exiting the kSep chamber was regularly sampled to monitor the formation and stability of the fluidized bed (Figure 6B). A fifth run was performed, but the formation of the fluidized bed was not monitored. In all four monitored runs, the fluidized bed formed within about eight min (Figure 6B). Across all five runs, the cell recovery was >90%, and any loss in viability was ≤1.3% (Table 3). It was possible to concentrate the cells up to 2.26 × 10^8^ viable cells/mL. After concentration, the cells from each run were plated onto 2D cell culture plates and were subjected to quality testing (see Section 2.9). After the first two runs, it was observed that there was an increasing percentage (up to an additional 5%) of cells escaping the fluidized bed at the end of the concentration process.

It is unlikely that this uptick was due to exceeding chamber capacity because the same pattern was observed in the run with the highest cell density as well as the run with the lowest. Regardless of the difference in cell number, the uptick was observed both in run 3 (~12 × 10^9^ hiPSCs in the chamber) and in run 1 (~3 × 10^9^ hiPSCs in the chamber). It could be due to cells settling in the feed bag, resulting in a sudden influx of concentrated cells (or cell clumps) that disturbed the fluidized bed.

### 2.9. Quality Assessment of hPSCs Concentrated after Expansion in Bioreactors

**Figure 7:**
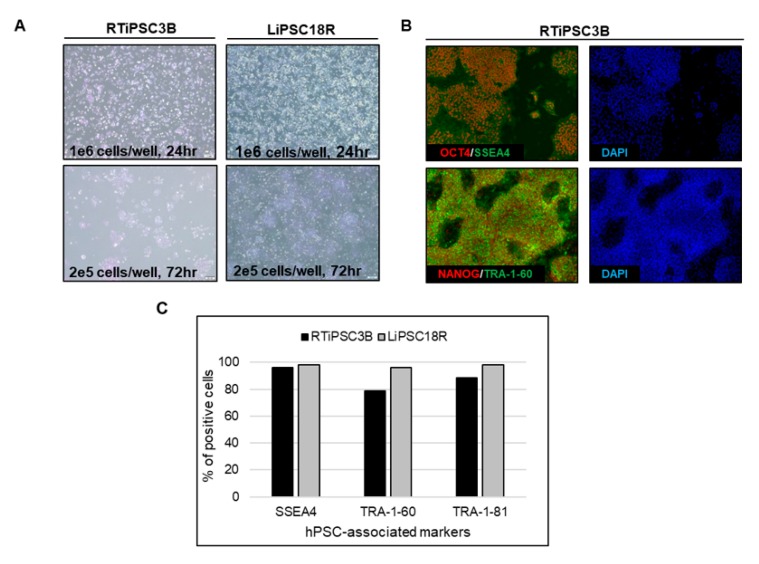

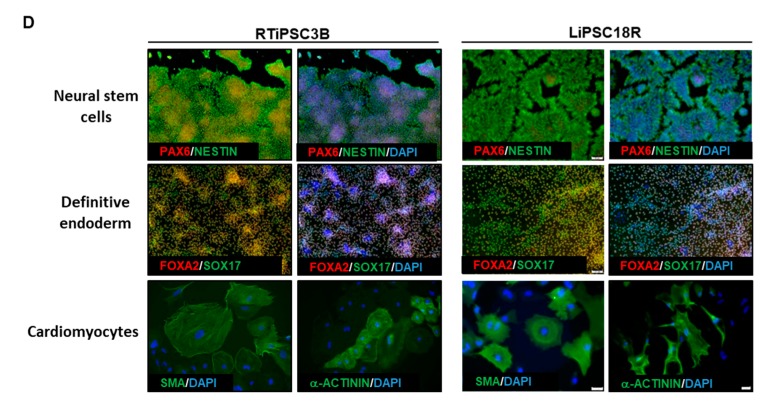
Cells expanded in a bioreactor and concentrated by kSep show hPSC characteristics of morphology and marker expression. (**A**) Phase contrast images of single cells (RTiPSC3B and LiPSC18R) post concentration by kSep400, 24 h and 72 h post plating onto 2D cell culture plates. Magnification, 40×; (**B**) Immunofluorescence staining RTiPSC3B cells expanded in a bioreactor and concentrated via kSep show expression of OCT3/4, NANOG, SSEA-4 and TRA-1-60. Magnification, 100×; (**C**) Quantitative analysis of hPSC-associated markers SSEA4, TRA-1-60 and TRA-1-81 by flow cytometry of iPSCs expanded in a bioreactor and concentrated via kSep. Results are shown for RTiPSC3B and LiPSC18R lines; (**D**) Pluripotency of RTiPSC3B and LiPSC18R cells expanded in a bioreactor and concentrated by kSep were demonstrated by directed differentiation into definitive endoderm, neural stem cells and cardiomyocytes. Magnification, 100× (Neural stem cells, definitive endoderm), 200× (cardiomyocytes)

Quality assessments of the expanded hiPSCs post kSep included cell attachment, morphology, hPSC-associated marker expression, karyotype and pluripotency. Post kSep, single cell hiPSCs (RTiPSC3B and LiPSC18R cell line) were plated in two different cell densities on 2D cell culture plates coated with the L7™ hPSC Matrix. The cells, cultured in L7™ TFO2 medium, attached well and grew as a monolayer when 1 × 10^6^ cells were seeded, while formation of small colonies were observed on day 3 when 2 × 10^5^ cells were seeded (Figure 7A). Likewise, the cells expressed the hPSC markers as determined qualitatively by immunofluorescence staining and quantitatively by flow cytometry (Figure 7B,C, respectively).

To determine whether cells concentrated by kSep are also capable of giving rise to all three germ layers, directed differentiation of RTiPSC3B and LiPSC18R cells post kSep was performed. As shown in Figure 7D, cells post expansion in a bioreactor, harvested as single cells and concentrated by kSep, were capable of directly differentiating into neural stem cells, as seen by positive staining for PAX6 and NESTIN; definitive endoderm, as shown by positive staining for FOXA2 and SOX17; and cardiomyocytes, as seen by positive staining for SMA and α-ACTININ, post contraction. The karyotype of LiPSC18R from two independent bioreactor runs, followed by kSep concentration, was determined to be normal (Table 1).

### 2.10. Quality Assessment of hPSCs Cryopreserved Following Expansion in Bioreactors and kSep Concentration

Cryopreservation of cell-based therapeutic products is a critical aspect of cell therapy [24,25,26]. Master and working cell banks of iPSCs could be readily used for subsequent rounds of expansion and differentiation into the desired cell therapy product. A key hurdle, however, is maintaining the viability and performance of cryopreserved cells [24].

Therefore, we checked whether bioreactor-expanded cells concentrated by kSep and which were also further cryopreserved, retain high viability and quality post thaw. 

**Figure 8:**
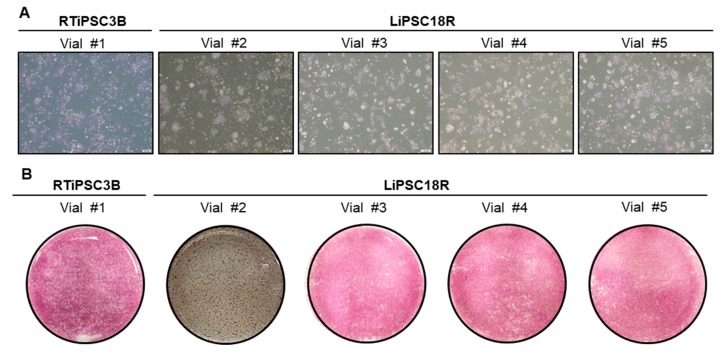
Quality of cryopreserved cells post thaw. (**A**) 6 × 10^5^ cells from each vial were plated onto a well of a 6-well plate. Phase contrast images were taken 48–72 h post thaw. Magnification, 40×; (**B**) Cells were stained with AP staining kit three (vial #2) to five (all other vials) days post plating. Whole-well images are shown. Magnification, 1×.

Human iPSCs expanded in a 3 L bioreactor and concentrated via kSep were cryopreserved in 1 mL of cryopreservation solution as described in the Materials and Methods. Cells at high viability of >85% were cryopreserved in various cell densities. The cryopreserved cells were thawed approximately two weeks after cryopreservation, and cell viability and vitality were determined. The viability of the thawed cells was similar across the various cryopreservation cell densities, however, lower than that before cryopreservation (Table 4). Upon plating onto 2D cell culture plates, cells attached, expanded and were positive for alkaline phosphatase staining (Figure 8A,B). This data also shows the feasibility to cryopreserve hiPSCs to a high cell density of 120 and 240 × 10^6^ cells/ml. This will shorten a 2D seed train for further processes involving cell expansion and differentiation.

Our results demonstrate that hiPSCs cryopreserved at high cell densities after harvest and subsequent concentration by kSep can be recovered successfully. The cells retain hPSC characteristics as shown by morphology upon plating in 2D and expression of the hPSC-associated marker, alkaline phosphatase. Therefore, we next examined whether the cryopreserved cells could be used to inoculate a 3 L bioreactor vessel (eliminating the need for a 2D seed train), while retaining their capability of self-renewal and differentiation.

### 2.11. Thaw to 3D Inoculation

Generating sufficient cells in a 2D seed train required in order to provide adequate inoculum for a larger bioreactor is time consuming, highly manual and involves a process vulnerable to contamination. Moreover, given the variability typically observed between the cell lines [27], culture of hPSCs by a 2D seed train depends on subjective decision making, and often requires highly-trained personnel, capable of monitoring for culture irregularities, which could adversely impact subsequent cell expansion in a bioreactor. In order to overcome these challenges, we tested whether a 2D seed train could be avoided by thawing cryopreserved cells into a suspension culture.

**Figure 9:**
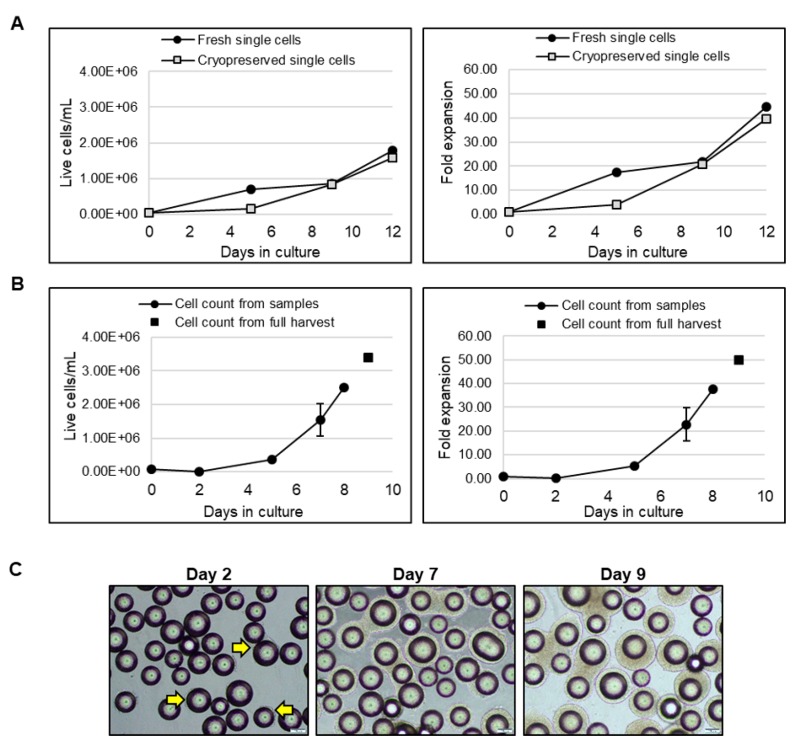
iPSCs thawed directly into suspension show high fold expansion. (**A**) Cell growth (left panel) and fold expansion (right panel) of directly thawed vs. freshly inoculated LiPSC18R in spinner flasks; (**B**) Cell growth (left panel) and fold expansion (right panel) of LiPSC18R thawed into a 3 L bioreactor; (**C**) Phase contrast images demonstrating cell growth on MCs on different days of the bioreactor run. Arrows indicate cells growing on MCs on day 2. Magnification, 100×; Scale bar: 100 µm.

**Figure 10:**
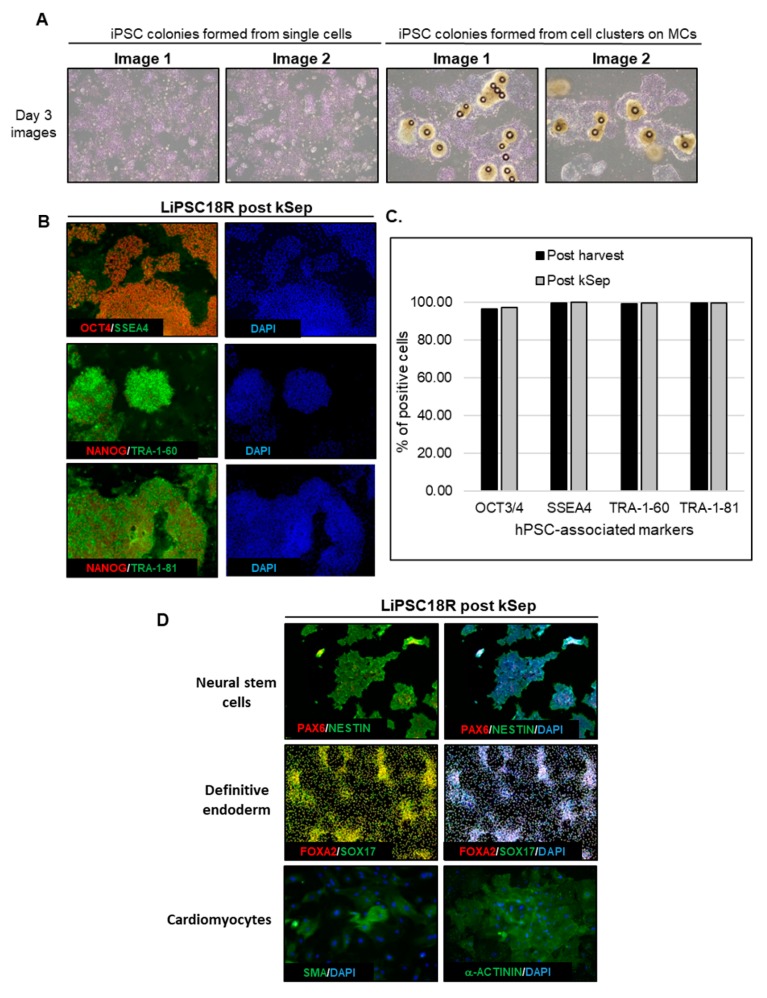
Thaw to 3D inoculation: iPSCs thawed into suspension retain hPSC-characteristics. (**A**) iPSCs thawed into suspension and expanded in a bioreactor have typical iPSC morphology when plated onto 2D, either before or after release from MCs. Magnification, 40×.; (**B**) Detection of hPSC-associated markers by immunofluorescence staining in cells post-harvest and concentration by kSep400. Magnification, 100×.; (**C**) Quantitative analysis of hPSC-associated markers by flow cytometry of cells post-harvest and cells concentrated after harvest; (**D**) Direct differentiation of iPSCs thawed into suspension, expanded in a bioreactor and concentrated via kSep400. Magnification, 100× (Neural stem cells, definitive endoderm), 200× (cardiomyocytes).2.13. D to 3D Inoculation.

LiPSC18R, cryopreserved as single cells, were thawed and inoculated into a spinner flask at a cell density of 0.04 × 10^6^ cells/mL. In parallel, 2D-cultured LiPSC18R cells were dissociated and inoculated as single cells into another spinner flask at the same cell density. Growth and expansion graphs demonstrate that cryopreserved and fresh single cell inoculum reach comparable cell density and fold expansion on day 9 (Figure 9A). To demonstrate the scalability of these findings, we inoculated a 3 L bioreactor with LiPSC18R cells previously expanded in a 3 L bioreactor, concentrated by kSep400 and cryopreserved (0.068 × 10^6^ cells/mL seeding density). Nine days after inoculation, a cell density of 3.5 × 10^6^ live cells/mL and total expansion >50-fold were achieved (Figure 9B). Representative phase contrast images show continued expansion of LiPSC18R on L7™ hPSC Matrix-coated MCs over time in suspension culture (Figure 9C). Following harvest, concentration and cryopreservation of these cells, we continued to assess the quality of expanded cells. Figure 10A (left panel) shows the cell attachment and growth as a monolayer culture when single cells were seeded and cultured. When cell-MC clusters were directly plated onto 2D culture plates, colonies with typical iPSC morphology were observed (Figure 10A, right panel). Moreover, representative immunofluorescence images and flow cytometry analyses confirmed expression of hPSC-associated markers (Figure 10B,C, respectively). Directed differentiation of these cells to the three germ layers was also confirmed by immunostaining for cell lineage-specific markers (Figure 10D). These results demonstrate that cryopreserved hiPSC inoculum is capable of expanding in a MC-based suspension system, resulting in a large number of high-quality hiPSCs.

Cell densities of >2 × 10^6^ hiPSCs/mL were achieved upon inoculation of 0.02–0.07 × 10^6^ cells/mL in a stirred tanked bioreactor (Table 5). As the volume of the bioreactor increases, the amount of inoculum needs to increase proportionally. Generating this inoculum in a traditional manual and open 2D process is non-ideal, with an increased risk of execution failures.

Our findings, which demonstrate the feasibility of inoculating 3 L bioreactors with cryopreserved cells and achieving approximately 50-fold expansion, show that a 2D seed train could be completely replaced by a closed 3D seed train. Nevertheless, to meet the required cell number for inoculation of 50 L or larger bioreactors, a considerably-sized working cell bank derived from 2D cell culture or the suspension culture system is still required. To circumvent this obstacle and mitigate challenges involved with the large scale manufacturing of hPSCs, we wanted to determine if a 3D seed train, i.e., the transfer of expanded cells from one suspension vessel to another to continue the expansion process, is feasible.

**Figure 11:**
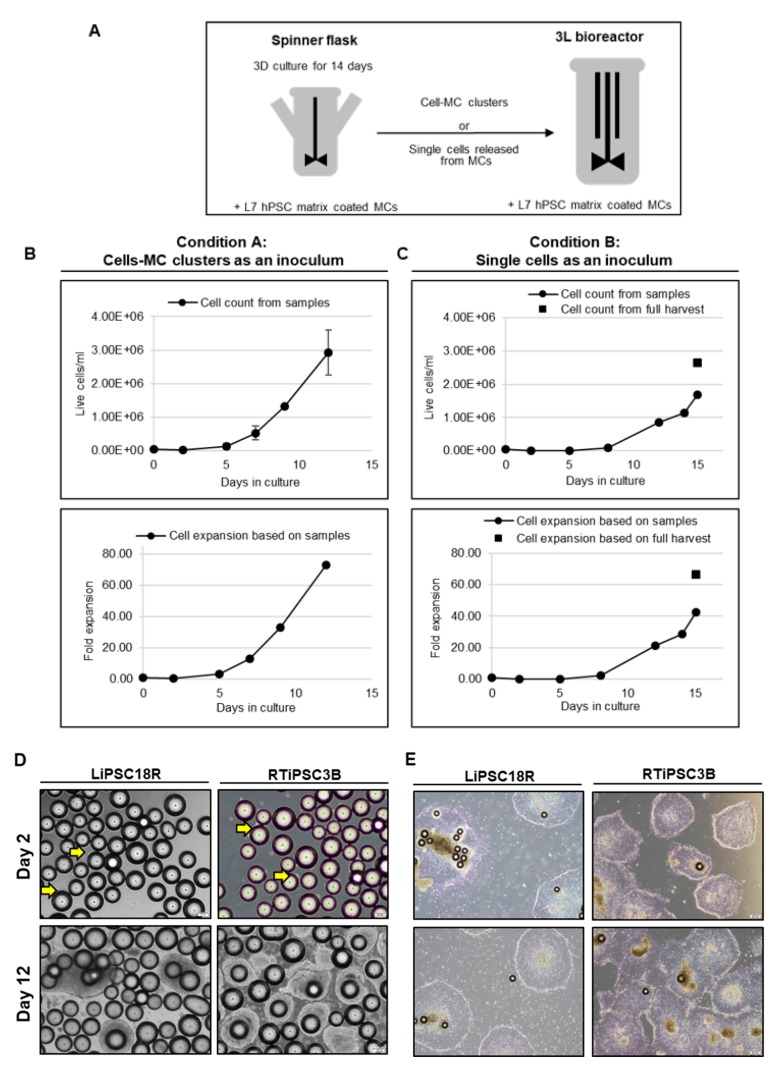
Three dimensional (3D) to 3D inoculation. (**A**) Schematic representation of experimental design for using the 3D seed train as inoculum; (**B**) Cell growth (upper panel) and fold expansion (lower panel) of LiPSC18R on MCs collected from a spinner flask and inoculated in a 3 L bioreactor; (**C**) Cell growth (upper panel) and fold expansion (lower panel) of RTiPSC3B released from MCs as single cells and inoculated in a 3 L bioreactor; (**D**) Phase contrast images of LiPSC18R and RTiPSC3B cells growing on MCs on different days in 3D culture. Arrows indicate cells growing on microcarriers on day 2. Magnification, 100×; Scale bar: 200 µm; (**E**) Phase contrast images of colonies formed by cells expanded in a bioreactor. Cells were plated as cell–MC clusters. Images are shown for LiPSC18R cells (3D seed train by transferring cell–MC clusters) and RTiPSC3B (3D seed train by transferring single cells). Images were taken five days post plating. Magnification, 40×.

To demonstrate the feasibility of a 3D seed train, two conditions were tested: re-inoculation of cell-MC clusters from a spinner flask to a 3 L bioreactor (Condition A, LiPSC18R cells) and transfer of single cells harvested from a spinner flask to a 3 L bioreactor (Condition B, RTiPSC3B cells) (Figure 11A). In both conditions, cell density at inoculation was 0.04 × 10^6^ cells/mL and cells were expanded with L7™ hPSC Matrix-coated MCs. Condition A resulted in a maximum cell density of 2.92 × 10^6^ LiPSC18R cells/mL, corresponding to >70-fold expansion in 12 days (Figure 11B), which is comparable to our results in Figure 3. This finding suggests that cells from the original cell-MC clusters were released into the medium, either as a result of cell division or by physical forces (agitation, collision of microcarriers and cell-MC clusters), and they can attach and colonize the MCs devoid of hPSCs. Condition B resulted in ~70-fold expansion of RTiPSC3B cells on day 15, as determined by cell counts from a full harvest (Figure 11C), but, interestingly, exhibited a longer ‘lag phase’ that may be attributable to inoculating as single cells rather than cell clumps. Representative phase images of cell-MC clusters sampled from the bioreactor on day 2 and day 12 of the run, showing cell expansion (Figure 11D). Figure 11E shows formation of colonies with typical morphology from single cells or cell clusters expanded on MCs, five days after harvest and plating onto 2D culture plates.

**Figure 12:**
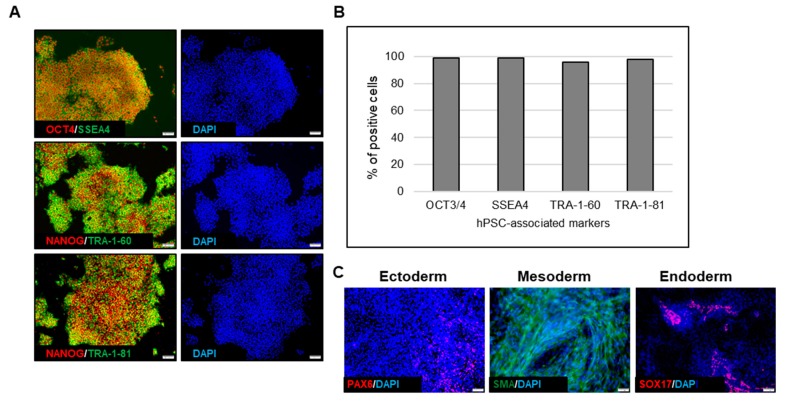
Quality assessment of hiPSCs expanded through a 3D seed train. (**A**) Immunofluorescence staining of hPSC-associated markers. Magnification, 100×; (**B**). Quantitative analysis of hPSC-associated markers; (**C**) Immunofluorescence staining of germ layer-specific markers, performed on embryoid bodies (EBs). Magnification, 100× (ectoderm, endoderm), 200× (mesoderm)

Additionally, representative immunofluorescence images (Figure 12A) showed the expression of hPSC-associated markers by RTiPSC3B cells expanded in a 3 L bioreactor through a 3D seed train (Condition B). Flow cytometry experiments confirmed that >90% of cells expressed OCT3/4, SSEA-4, TRA-1-81 and Tra-1-60 (Figure 12B). Embryoid body formation assay showed that these cells have retained hPSC differentiation potential (Figure 12C). Based on the results described above, we were able to successfully demonstrate that a 3D seed train could lead to high fold expansion of high quality cells, paving the way to commercial scale manufacturing of hPSCs.

## 3. Discussion

Stem cell technology has revolutionized regenerative medicine, ushering in a new era focused on curative therapies rather than disease management. Over the past decade, efforts toward the development and optimization of cGMP compliant, large-scale manufacturing of cell-based therapies have significantly increased [15,28]. Based on internal calculations, expansion of hPSCs in a scalable bioreactor leads to a cost reduction of 43% compared to the conventional 2D culture method. In this study, we developed a microcarrier-based bioreactor suspension platform to expand hiPSCs to cell densities of >2 × 10^9^ cells/L using xeno-free, fully defined hPSC medium with a closed, automated process for hiPSC harvest and concentration, and extensively characterized the expanded hiPSCs.

Our proprietary hPSC culture system, which includes the L7™ TFO2 hPSC medium and matrix, supported the expansion of the hiPSCs tested in manually operated, open, spinner flasks and in automated, closed, stirred tank bioreactors. Feasibility experiments performed in spinner flasks inoculated with 0.2 × 10^6^ cells/mL and microcarriers resulted in 10-fold expansion within 17 days without the need for cell passaging. Results were comparable to those previously published when assessing hESC growth on laminin-coated MCs [19]. However, in our study the cells did not require adaptation to suspension culture by pre-conditioning with the MCs in static culture conditions; rather, they could be inoculated directly.

In addition to the composition of the medium in which hPSCs are expanded, optimizing cell inoculum density is a critical factor in hiPSC expansion in suspension systems [29]. Our findings, which compared lower and higher inoculum cell densities, support a growing body of evidence that higher fold expansion can be achieved using lower seeding densities [21,29]. Moreover, seeding density not only impacts the rate and quality of expansion, but also the cost, labor and expenditure of time associated with achieving the necessary cell density at inoculation.

In light of our findings, we adapted our process and demonstrated scalability in 1 L or 3 L bioreactor vessels. We successfully produced 6–15 × 10^9^ cells per 3 L bioreactor run, (cell line and harvest day-dependent) meeting the number of cells required for a number of clinical indications [29]. Over 90-fold expansion was achieved within 9–16 days when using 2D cultured cells as bioreactor inoculum, which is a higher fold expansion than those achieved in spinner flask cultures. This is likely attributable to a better control of key parameters, which influence the rate of hiPSC expansion. The continuous media change, achieved through perfusion, facilitates control of key nutrients and metabolites, such as glucose and lactate. pH was controlled via a one-sided control regime in which CO_2_ was used to reduce pH when it drifted above the set point of 7.2. Figure 4C shows that this prevented the pH from rising more than 0.1 units above the set point. However, there was no active control to raise pH (such as base addition), only the passive gradual increase in pH brought about by off-gassing of CO_2_. Therefore, as cells expanded, pH dropped gradually to ~6.8’.

Upon increase in cell expansion, however, we observed a reduction in DO levels, which could not be maintained at the set target of 50%, and reached 10% at cell densities >3 × 10^6^ cells/mL. Previous studies have demonstrated that O_2_ levels regulate hPSCs’ metabolic flux, but the expression of pluripotency and differentiation markers in hPSCs cultured in either 20% or 5% O_2_ was unaltered. Moreover, no differences in proliferation were observed, suggesting that low O_2_ improves hPSC stemness [30,31,32]. Others have also shown that 30% DO is the optimal condition to support hPSC expansion [33]. In line with this study, we did not observe an adverse impact on the quality of cells harvested at cell densities of ~2.5 × 10^6^ cells/mL, where DO levels remained >30. Moreover, characterization of cells harvested from a 3 L bioreactor at a cell density of >5 × 10^6^ cells/mL, with corresponding DO levels of 10%, showed that the cells had a normal karyotype, expressed hPSC-associated markers, and were capable of differentiating into cells of the three germ layers. These findings suggest the minimal impact of O_2_ levels on the quality of cells expanded in our platform.

To increase cGMP process compliance, we demonstrated that cells expanded in stirred tank bioreactors could be harvested and concentrated in a closed and automated manner. To date, only one other report exists of concentrating hPSCs using kSep, concentrating ~1.2 × 10^9^ hPSCs tenfold, with 65% recovery of viable cells [34]. In our process, on average, the kSep process retained 94% of all harvested cells, and was able to process a 3 L bioreactor in under 30 min, concentrating cells up to 105-fold. Our data show that at least 48 × 10^9^ hPSCs can be harvested per kSep 400 cycle (12 × 10^9^ cells per chamber × four chambers), though further experiments would be needed to find the maximum capacity. This maximum capacity will be the main scale-up constraint: while the 120 mL/min flow rate could reasonably process a 50 L bioreactor in 110 min, the total cells harvested from such a bioreactor (100 × 10^9^) could potentially exceed the kSep 400 capacity. This could be overcome by processing two kSep units in parallel, or by harvesting the cells from one unit over two sequential cycles.

The seamless implementation of a closed, automated concentration step using the kSep400, and subsequent recovery of high-quality hiPSCs, demonstrate the adaptability of our platform. The development of a robust, flexible and adaptable process is needed, given the continued evolution of cGMP policies and innovations in large-scale manufacturing. Therefore, our MC-based and downstream processing compatible platform allow large-scale production of hiPSCs without compromising the quality of expanded hiPSCs.

Another key element in the large-scale manufacturing of cell-based therapies is maintaining the viability of cryopreserved cells, such that the therapeutic potential of these cells remains intact [25]. We extensively characterized the cryopreserved hiPSCs upon thaw, and confirmed their quality.

In the interest of cost effectiveness and mitigation of risks such as contamination, we demonstrated the ability to directly inoculate cryopreserved cells into 3D. Cryopreserved cells used for the inoculum showed high fold expansion and quality, as was confirmed by cell morphology, expression of hPSC-associated markers and the ability to directly differentiate. Further development of our platform shows the feasibility of a 3D seed train such that cells expanded in a 3 L bioreactor, for example, could potentially be reinoculated in larger bioreactor vessels. Assuming a conservative cell number of 2 × 10^9^ cells/L, cells from one 3 L bioreactor could be potentially serve as the inoculum for a 3 × 50 L bioreactor. Assuming an even higher cell yield, achievable by a longer culture period in the bioreactor, one 3L could serve as an inoculum even for several 50 L bioreactors or one 250 L bioreactor. Together, this makes possible an expansion process which is completely 2D-free, closed, automated and requires minimal labor. This will enable the commercialization of clinical indications that require large cell numbers, consequently increasing the availability of cell-based therapies. Further, our end-to-end platform has the potential to support the scalable induction of disease-relevant cells from hPSCs, and their subsequent expansion which is critical in the development of commercially ready therapies.

## 4. Materials and Methods

### 4.1. L7™ hPSC Culture System

The Lonza L7™ culture system was developed specifically for culturing human embryonic stem cells (hESCs) and human-induced pluripotent stem cells (hiPSCs) in a feeder-free environment [35]. This culture system allows feeding on an every-other-day media change schedule. It is comprised of recombinant, xeno-free and defined L7™ hPSC Matrix (Lonza, FP-5020, Walkersville, MD, USA) to enable cell attachment, xeno-free L7™ hPSC basal medium, xeno-free L7™ hPSC medium supplement and non-enzymatic passaging solutions: L7™ hPSC passaging solution (yields cell clumps, Lonza, FP-5013) or F3 hPSC passaging solution (yields single cells). The L7™ hPSC basal medium used in the work described here was modified by replacing native, animal-based components with respective recombinant components. It is referred to as L7™ TFO2 hPSC basal medium.

### 4.2. Human iPSC Lines

The human LiPSC18R induced pluripotent stem cells (iPSC) line was generated from CD34+ cord blood cells as previously described [36]. RTiPSC3B and RTiPSC4i were generated from human peripheral blood mononuclear cells (PBMNCs, Lonza, CC-2702, Walkersville, MD, USA) of two different donors. Cryopreserved PBMNCs were thawed and cultured for six days in a priming medium comprised of an animal-free Hematopoietic Progenitor Growth Medium (HPGM™) (equivalent to Lonza, PT-3926, Walkersville, MD, USA, where native components were replaced with the respective recombinant ones), supplemented with 100 ng/mL recombinant human stem cell factor (rhSCF) (PeproTech, AF-300-07, Rocky Hill, NJ, USA), 40 ng/mL insulin-like growth factor-1 (IGF-1) (PeproTech, AF-100-11, Rocky Hill, NJ, USA), 10 ng/mL IL-3 (PeproTech, AF-200–03), 1 µM dexamethasone (Sigma, D1756, St. Louis, MO, USA), 100 µg/mL holo-transferrin (R&D Systems, 2914-HT, Minneapolis, MN, USA) and 200µM 1-thioglycerol (Sigma, M6145, St. Louis, MO, USA). The PBMNCs were seeded in 6-well plates (Corning, 353046, corning, NY, USA) at a density of 2–4 × 10^6^ cells/mL. On day 3, cells were collected, counted and seeded in a fresh priming medium at a density of 0.5–1 × 10^6^ cells/mL. On day 6, cells were collected and cellular reprogramming was performed.

To reprogram the cells, 1 × 10^6^ PBMNCs were nucleofected with the episomal plasmids pCE-hOCT3/4, pCE-hSK, pCE-hUL, pCE-mP53DD and pCXB-EBNA-1 [37]. Nucleofection was performed using the 4D-Nucleofector™ System and P3 solution Kit (Lonza, V4XP-3012). After nucleofection, the cells were plated onto 6-well plates pre-coated with L7™ hPSC Matrix, in priming medium containing 0.5 mM sodium butyrate (ReproCELL USA, Inc., 04-0005, Beltsville, MD, USA) to enhance reprogramming efficiency. Plates were placed in a 37 °C humidified incubator (5% CO_2_ and 3% O_2_). Two days post plating, L7™ hPSC medium was added to the wells without removing the priming media (1:1 ratio). On day 4, the media was aspirated and fresh L7™ hPSC medium containing 0.5 mM sodium butyrate was added. Media change was performed in an every-other-day manner, and cells were incubated in a 37 °C humidified incubator (5% CO_2_ and 3% O_2_) until hiPSC colonies formed and were picked for further expansion and characterization. Colony picking was performed mechanically, while subsequent cell passaging was performed with L7™ hPSC passaging solution (yields cell clumps, Lonza, FP-5013). These iPSC lines were characterized, showing normal karyotype, expression of key hPSC-associated markers, and demonstrating the potential to differentiate to cells of the three germ layers.

### 4.3. Culturing hiPSCs in 2D

Human iPSCs were cultured in 2D for the purpose of expanding cells for inoculation in a suspension vessel (spinner flask or stirred tank bioreactor) and characterization following expansion in suspension. hiPSCs were cultured in Lonza L7™ hPSC culture system using the animal-free L7™ TFO2 media and xeno-free L7™ hPSC media supplement. Cells maintained in culture were passaged at a 1:6 split ratio and allowed to reach confluence after 4–5 days depending on the cell line used. The cells were then harvested either as cell clumps by L7™ hPSC passaging solution (Lonza, FP-5013, Walkersville, MD, USA) or as single cells with F3 passaging solution to yield single cells and supplemented with 10 µM Y27632 (ReproCELL USA, Inc., 04-0012, Beltsville, MD, USA) at plating.

For the culture of the 2D seed train prior to inoculation in a 3 L bioreactor, human iPSCs were thawed and plated onto a L7™ hPSC matrix-coated T-75 culture flask and maintained in L7™ TFO2 media at a cell density of 0.02–0.04 × 10^6^ live cells/cm^2^. When the 2D culture reached 70–80% confluence, iPSC colonies were dissociated into cell clumps using the L7™ hPSC passaging Solution and plated onto a 1-layer CellStack (Corning, 05-539-094, corning, NY, USA) at a cell density range of 0.02–0.03 × 10^6^ live cells/cm^2^. When the 2D culture reached 70–80% confluence, iPSC colonies were dissociated into cell clumps using the L7™ hPSC Passaging solution and 62–120 × 10^6^ live cells were inoculated in a 3 L bioreactor. The NucleoCounter NC-200 (Chemometec, Copenhagen, Denmark) was used to assess the number and viability of the bioreactor cell inoculum.

### 4.4. Microcarrier Coating

Plastic microcarriers (MCs) (SoloHill brand polystyrene 90–150 µm, Pall Corporation, P-215-020 or 125–212 µm, Pall Corporation, P-221-020) were suspended in Dulbecco’s Phosphate-Buffered Saline with calcium and magnesium (DPBS+/+, Lonza, 17-513F) and coated with L7™ hPSC Matrix. MCs and L7™ hPSC Matrix were incubated for 2 hours at 37 °C. The DPBS+/+ solution was then aspirated, and the MCs were re-suspended in L7™ TFO2 hPSC basal medium and allowed to incubate overnight at room temperature with agitation. For expansion in 125 mL spinner flasks, 600 mg of MCs were incubated with 540 µg of L7™ hPSC Matrix. For expansion in a 3 L stirred tank bioreactor, 20 g of MCs were incubated with 18 mg of L7™ hPSC Matrix.

### 4.5. Culturing hiPSCs in a Spinner Flask

Human iPSCs were harvested from 2D as cell clumps or thawed directly as single cells into a 125 mL spinner flask (Corning, 3152, Corning, NY, USA), containing 100 mL L7™ media and L7™ hPSC Matrix-coated microcarriers (MCs). hiPSCs cultured in 2D were passaged with either L7™ hPSC Passaging solution to generate cell clumps or F3 passaging solution to dissociate colonies to single cells. The spinner flask was inoculated with either 0.04 × 10^6^ cells/mL of cell clumps or cryopreserved single cells. If single cells were inoculated, media was supplemented with 10 µM Y27632 (ReproCELL USA, Inc., 04-0012, Beltsville, MD, USA). Spinner flask cultures were incubated in a 37 °C humidified incubator containing 5% CO2 overnight. After 24 h, the spinner flask was placed on a magnetic stirring plate with an agitation speed of 25 RPM. The agitation speed was increased as needed to ensure that these hiPSC-MCs remain in suspension. The maximum agitation speed applied to support cell densities >2 × 10^6^ cells/mL was 90 RPM. Media was changed in an every-other-day manner using L7™ TFO2 media with xeno-free L7™ hPSC media supplement. To determine the growth of hiPSCs in culture, 5 mL samples were obtained and hiPSCs were dissociated from the microcarriers using the F3 hPSC passaging solution to generate single cells. The NucleoCounter NC-200 (Chemometec, Copenhagen, Denmark) was used to determine cell number and viability.

### 4.6. hPSC Expansion in Stirred Tank Bioreactors

The BioBlu single-use bioreactor vessel was set up according to manufacturer’s instructions (Eppendorf, 1386000300, Hauppauge, NY, USA). Briefly, the 3 L vessel was equipped with probes required for the online monitoring (Mettler Toledo, Columbus, OH, USA) of key parameters, including percentage of dissolved oxygen (DO), pH and temperature. The bioreactor was controlled using a G3 Lab Universal controller (Thermo Fisher Scientific, Waltham, MA, USA). Prior to inoculation, L7™ hPSC Matrix-coated plastic microcarriers were introduced and the vessel was calibrated as previously described [16] with L7™ TFO2 medium supplemented with L7™ hPSC medium supplement. The 3 L vessel was inoculated at day 0 with either 62–120 × 10^6^ 2D cultured cells in cell clumps (0.02–0.04 × 10^6^ cells/mL) or 204 × 10^6^ cryopreserved single cells at 37 °C with the initial agitation rate set to 50 RPM. To demonstrate the feasibility of a 3D seed train, 120 × 10^6^ cells from cell clusters grown on MCs, or single cells obtained post detachment from the cell-MC clusters, were inoculated into a 3 L bioreactor containing L7™ hPSC Matrix-coated plastic microcarriers (Figure 11A). On day 1, perfusion with fresh L7™ TFO2 supplemented medium was initiated at a rate of one Vessel Volume per Day (VVD). Perfusion was performed using a proprietary designed microcarrier retention filter. To monitor the changes in key metabolites, 5 mL samples were taken from the bioreactor at various time points along the run. Offline monitoring to determine changes in parameters such as pH and key nutrients was performed using the BioProfile FLEX Analyzer (Nova Biomedical, Waltham, MA, USA). To determine cell growth and fold expansion, 15 mL samples were taken in duplicates at various time points along the run, and hiPSCs were dissociated from the microcarriers using the F3 hPSC passaging solution to generate single cells. The Nucleocounter NC-200 (Chemometec, Copenhagen, Denmark) was used to measure the cell number and viability.

### 4.7. Harvest of hiPSCs from Stirred Tank Bioreactor

Human iPSCs in the 3 L bioreactor were collected upon reaching approximately >2 × 10^6^ cells/mL. With continuous agitation, the medium was first removed from the vessel. Warm F3 passaging solution was introduced to the vessel with continuous agitation. To verify detachment of hiPSCs from microcarriers, a 5 mL sample was obtained after 25–30 min of incubation with F3 passaging solution. The solution containing single cell hiPSCs and microcarrier was then transferred through a 30–65 µM pore sized filtration bag (Flex Concepts, FCC03475.01, Logan, UT, USA). The single cells were ultimately transferred into a 3 L bag containing L7™ TFO2 medium supplemented with L7™ hPSC medium supplement. Harvested cells were subjected to various assays including performance evaluation, characterization, downstream processing and cryopreservation. In bioreactor runs where the intent was to concentrate and cryopreserve the cells for future use, 10 µM Y27632 (ReproCELL USA, Inc., 00-0055, Beltsville, MD, USA, 04-0012) was added to the L7™ TFO2 media after treatment with F3 passaging solution.

### 4.8. Downstream Processing: Flow Rate Optimization for Fluidized Bed Formation

A kSep (Sartorius) was fitted with a 400.50 rotor, which functions as a 1/3.5 scale-down model for the kSep400. The associated 400.50 single-use kits (chamber set and valve set) were then installed. A 3 L PSC suspension was harvested from a bioreactor and connected as the feed source. A solution of PlasmaLyte-A (Baxter) and (0.25%) human serum albumin (Octapharma, Lachen, Switzerland) was used to prime the system and wash the cells. A centrifugation speed of 782× *g* was used. To optimize the formation of the fluidized bed, three flow rates (25, 30, 35 mL/min) were tested in increasing order. Prior to each run, the feed source was sampled in triplicate to determine cell density going into the kSep. For the entirety of the concentration process, 5 mL samples were drawn from the stream exiting the kSep chamber and tested using the NucleoCounter NC-200 (Chemometec, Copenhagen, Denmark) to monitor the number of cells escaping the fluidized bed. After 1 L of cell suspension was processed, the kSep was stopped, the chamber was emptied, and the concentrated cells were collected. The kSep was reset, the tubing and chambers were purged, and the process was repeated until all flow rates had been tested and the feed source was depleted.

### 4.9. Downstream Processing: hPSC Concentration Post Full Harvest 

A bag containing the filtered PSC suspension harvested from the bioreactor was sampled in triplicate, and the viabilities and cell densities were then determined using a NucleoCounter NC-200. The average viable cell density (VCD) was used to calculate the concentrated volume that would be harvested by the kSep (Equation (1), see Appendix A). The kSep400 (Sartorius) was equipped with its respective single-use kits (chamber set and valve set). A 10 L bag of DPBS (−/−) (Lonza) was used to prime the system (no wash steps were performed). The bag (the feed) was then welded onto the kSep valve set. The process recipe primed the system, then pumped cell suspension into one chamber at a rate of 120 mL/min (3.5× the value determined in the optimization experiment, rounded down). The process was run at 1000× g. These settings were maintained until the entirety of the feed was processed by the kSep. Periodically throughout the process, 5 mL samples were drawn from the stream exiting the kSep chamber and were tested using the NucleoCounter NC-200 to monitor the number of cells escaping the fluidized bed. After the feed bag emptied, the concentrated cells were harvested. The volume of the concentrate was verified, and samples were taken to determine viability and cell density. The remaining concentrate was cryopreserved.

### 4.10. Cryopreservation

Human iPSCs were suspended in cryopreservation solution (CS10, Biolife Solutions Inc., 210102, Bothell, WA, USA) containing 10 µM of Y-27632 (ReproCELL USA, Inc., 04-0012, Beltsville, MD, USA). Cryovials were cryopreserved by the Cryomed™ Controlled-rated Freezer (Thermo Fisher Scientific, Model 7456, Waltham, MA, USA) and subsequently stored in liquid nitrogen until use.

### 4.11. Immunofluorescence Staining

Cells cultured in 2D were fixed with 4% paraformaldehyde (Santa Cruz, SC 281692, Dallas, TX, USA) blocked with a blocking solution comprised of 10% donkey serum and 0.1% Triton X-100 in PBS −/−. The cells were incubated with primary antibodies followed by secondary antibody incubation and 4′,6-diamidino-2-phenylindole (DAPI) staining. Immunofluorescence was observed using an Olympus IX73 microscope. The following primary antibodies were used to detect hPSC-associated markers: OCT4/POU5F1 (Abcam, ab19857, Cambridge, UK), NANOG (R&D systems, AF1997, Minneapolis, MN, USA), TRA-1-81 (ReproCELL USA, Inc., 09-001, Beltsville, MD, USA), TRA-1-60 (Millipore, MAB4360, Burlington, MA, USA) and SSEA-4 (Millipore, MAB4304, Burlington, MA, USA). The following primary antibodies were used to detect expression of germ-layer specific markers: SOX17 (R&D systems, AF1924, Minneapolis, MN, USA), FOXA2 (Abcam, Ab108422, Cambridge, UK), NESTIN (R&D systems, MAB1259, Minneapolis, MN, USA), PAX6 (Biolegend, #901301), α-actinin (Sigma, A7811, St. Louis, MO, USA) and SMA (Millipore, CBL171, Burlington, MA, USA).

### 4.12. Flow Cytometry

Quantitative detection of hPSC-associated markers was performed using flow cytometry as previously described [16,38,39]. Briefly, single cells were live-stained for the cell surface markers: TRA-1-81 (BD Biosciences, #560161, San Jose, CA, USA), TRA-1-60 (BD Biosciences, #560884) and SSEA-4 (BD Biosciences, #560126, San Jose, CA, USA). Cells were also fixed, permeabilized and stained for OCT4/POU5F1 (Cell Signaling, #5177S, Danvers, MA, USA). The samples were processed using either FACSCanto^TM^ II (Becton Dickinson) or the FACSCelesta^TM^ (Becton Dickinson, San Jose, CA, USA), and data was acquired using the BD FACSDiva Software followed by analysis using FlowJo v10 software (FlowJo, San Jose, CA, USA).

### 4.13. Alkaline Phosphatase Staining

Alkaline phosphatase staining was performed using StemAb Alkaline Phosphatase Staining Kit II (ReproCELL USA, Inc., 00-0055, Beltsville, MD, USA), following manufacturer instructions.

### 4.14. Karyotyping

Live cells were plated onto a T-25 flask, pre-coated with L7™ hPSC Matrix and maintained in L7™ TFO2 hPSC medium. Karyotype analysis (G-banding) was performed at LabCorp (Santa Fe, New Mexico).

### 4.15. Embryoid Body Formation

Embryoid body (EB) formation was performed by plating single cells in AggreWell800 (Stem Cell Technologies, 34811, Vancouver, Canada) in medium containing Knockout DMEM F-12 (Gibco,12660-012, Grand Island, NY, USA), 20% Knockout Serum (Gibco, 10828-028, Grand Island, NY, USA), non-essential amino acids-1x (Gibco, 11140-050) and Glutamax-1x (Gibco, 35050-061). Medium was changed 48 hours later and thereafter till day 7 in an every-other-day mode. On day 7, EB spheres were collected and plated onto plates coated with 0.1% Gelatin (Millipore, ES-006-B) and medium containing Dulbecco’s modified Eagle’s medium (DMEM) (Gibco 11965-092), 20% fetal bovine serum (FBS) (Gibco, SH30071), non-essential amino acids-1x (Gibco, 11140-050) and Glutamax-1x (Gibco, 35050-061). Medium was changed every other day for 7 days. On day 7 post plating, EBs were fixed with 4% paraformaldehyde (Santa Cruz, SC-281692, Dallas, TX, USA), and stained for detection of cells of the three germ layers with antibodies for the following antigens: SOX17 (R&D Systems, AF1924, Minneapolis, MN, USA) for endoderm, PAX6 (BioLegend, PRB-278P, San Diego, CA, USA) for ectoderm and SMA (Millipore, CBL171) for mesoderm.

### 4.16. Definitive Endoderm Differentiation

Human iPSCs were differentiated into definitive endoderm (DE) as previously described [39]. Briefly, 0.25 × 10^6^ single cells were seeded onto L7™ hPSC Matrix-coated 24-well plates on day 0 with L7™ TFO2 media containing L7™ hPSC medium supplement and 10 µM Y27632 (ReproCELL USA, Inc., 04-0012, Beltsville, MD, USA). On day 1, the STEMdiff™ Definitive Endoderm Kit (Stem Cell Technologies, 05110, Vancouver, Canada) was used to induce DE differentiation according to manufacturer’s protocol.

The cells were washed, fixed on day 5 and stained for DE-specific markers SOX17 (R&D systems, AF1924, Minneapolis, MN, USA) and FOXA2 (Abcam, Ab108422, Cambridge, UK).

### 4.17. Neural Stem Cell Differentiation

Human iPSCs were differentiated into neural stem cells (NSCs) as previously described [39]. In brief, 0.25 × 10^6^ single cells were seeded onto L7™ hPSC Matrix-coated 6-well plates on day 0 with L7™ TFO2 media containing L7™ hPSC medium supplement and 10 µM Y27632 (ReproCELL USA, Inc., 04-0012, Beltsville, MD, USA). On day 1, the culture medium was changed with Neural Induction Medium (NIM) composed of B-27 Plus Neuronal Culture System (Gibco, A3653401) with 1X Glutamax (Gibco, 35050-061), 4 µM CHIR99021 (ReproCELL USA, Inc., 04-0004-02, Beltsville, MD, USA), 3 µM SB431542 (ReproCELL USA, Inc. 04-0010-10, Beltsville, MD, USA) and 10 ng/mL hLIF (Peprotech, 300-00-250, Rocky Hill, NJ, USA). NIM was changed in an every-other-day manner. When the cells reach 95–100% confluence, the cells were passaged as single cells using the F3 passaging solution. 1 × 10^6^ and 0.25 × 10^6^ cells were seeded onto 6- and 24- well plates, respectively (NSC-P1). NIM was replenished the following day and every-other-day till the cells were fixed and stained for neural progenitor markers, NESTIN (R&D systems, MAB1259, Minneapolis, MN, USA) and PAX6 (Biolegend, 901301, San Diego, CA, USA). The cell culture plates used for NSC culture were pre-coated by incubating with 20 g/mL Poly-l-ornithine (Sigma P4957, St. Louise, MO, USA) in sterile cell culture grade water (Lonza 17-524F, Wallkersville, MD, USA) for 2 h at 37 °C. The plates were then washed with calcium and magnesium free DPBS (DPBS-/-) (Lonza, 17-512F, Wallkersville, MD, USA), followed by a 1 hour incubation at 37 °C with 15 g/mL Laminin (Sigma, 11243217001, St. Louis, MO, USA) re-suspended in DMEM/F12 (Gibco, 11330032, Grand Island, NY, USA) or PBS −/− (Lonza, 17-516F, Wallkersville, MD, USA).

### 4.18. Cardiac Differentiation

Human iPSCs were differentiated into cardiomyocytes using the Gsk3 inhibitor and Wnt inhibitor (GiWi) protocol as previously described [5,40,41]. Briefly, 1 × 10^6^ single cells/mL were seeded onto L7™ hPSC Matrix-coated 6-well plates in the presence of 10 µM Y27632 (ReproCELL USA, Inc., 04-0012, Beltsville, MD, USA). Cells were maintained with L7™ TFO2 media containing L7™ hPSC medium supplement until confluence, during which time the cells were treated with 6–12 µM CHIR99021 (Tocris Bioscience, 4423, Bristol, UK) in RPMI/B27-insulin medium (day 0). After 24 hours, the medium was changed to fresh RPMI/B27-insulin (day 1). 5–7.5 µM IWP2 (Tocris Bioscience, 3533, Bristol, UK) was added on day 3, with fresh medium added on day 5. From day 7, cells were maintained in RPMI/B27 medium with medium changed in an every-other-day manner until spontaneous contraction was observed. Thereafter, iPSC-derived cardiomyocytes were dissociated as single cells using 10X TrypLE™ Select Enzyme (Gibco, 12563011, Grand Island, NY, USA) for 5–10 min at 37 °C. Cells were plated on 24-well plates coated with 0.1% gelatin (Millipore, ES-006-B) in EB20 medium, which is comprised of DMEM/F12 (Gibco, 11330032, Grand Island, NY, USA), FBS (GE Healthcare, SH30071.01, Chicago, IL, USA), modified Eagle’s medium (MEM) Non-Essential Amino Acids (Thermo Fisher Scientific, 11140050, Waltham, MA, USA), GlutaMAX™ Supplement (Gibco, 35050061, Grand Island, NY, USA) and 2-Mercaptoethanol (Thermo Fisher Scientific, 21985023, Waltham, MA, USA). The cells were fixed and stained for mesoderm-specific markers, α-ACTININ (Sigma, A7811, St. Louis, MO, USA) and smooth muscle actin (SMA) (Millipore, CBL171, Burlington, MA, USA).

## 5. Conclusions

L7™ TFO2 hPSC xeno-free medium supports hiPSC microcarrier-based expansion in stirred tank bioreactors.

Inoculum cell density of 0.02–0.06 × 10^6^ cell/mL is sufficient to yield >6–9 × 10^9^ cells/L in 9–14 days.

Expanded cells could be efficiently concentrated by automated centrifugation without compromising cell viability, yield and quality.

Expanded cells could be cryopreserved at high cell densities, without compromising cell viability and quality.

A 2D seed train could be avoided by using cryopreserved cells for inoculation.

A 3D seed train could be performed, allowing transfer of cells from one vessel to another and support up scaling of the expansion process.

## 6. Patents

The topics, ideas and concepts discussed herein may be covered by one or more patents or patent applications. For further reference of patents owned and/or controlled by Lonza, please visit www.lonza.com

## Figures and Tables

**Table 1:** Summary of karyotyping results of cells expanded either in 1 L- or 3 L-stirred tank bioreactors. Upon harvest from the bioreactor, cells were plated on T-25 flasks for karyotyping before or after kSep concentration. Cells were cultured in 2D for 1–10 passages, thus showing the karyotype integrity of cells expanded in a bioreactor.

Cell Line	Passage Number at Inoculation	Bioreactor Size	# of 2D Passages Post-Harvest	Cell Processing before Karyotyping	Karyotype
RTiPSC3B	P16	1L	P1	Expansion and harvest	Normal
RTiPSC3B	P16	1L	P1	Expansion and harvest	Normal
RTiPSC4i	P28	1L	P1	Expansion and harvest	Normal
RTiPSC4i	P28	1L	P10	Expansion, harvest and culture in 2D	Normal
RTiPSC3B	P22	3L	P2	Expansion and harvest	Normal
RTiPSC3B	P28	3L	P1	Expansion and harvest	Normal
RTiPSC4i	P31	3L	P1	Expansion and harvest	Normal
RTiPSC3B	P28	3L	P1	Expansion and harvest	Normal
RTiPSC3B	P28	3L	P1	Expansion and harvest	Normal
LiPSC18R	P26	3L	P1	Expansion and harvest	Normal
LiPSC18R	P29	3L	P1	Expansion, harvest and concentration	Normal
LiPSC18R	P34	3L	P1	Expansion, harvest and concentration	Normal

**Table 2:** Cell counts and viability before and after kSep concentration; v c, viable cell number; c, cells.

		Before kSep			After kSep				
Flow Rate (mL/min)	Harvest Volume (mL)	Viable Cells into kSep (v c)	Viable Cell Density (c/mL)	Viability (%)	Viable Cells Harvested (v c)	Viable Cell Density (c/mL)	Viability (%)	Total Cells Harvested (c)	Recovery (%)
**30**	25	3.20 × 10^9^	2.94 × 10^6^	87.60	2.55 × 10^9^	1.02 × 10^8^	90%	2.84 × 10^9^	80%
**35**	30	3.19 × 10^9^	3.11 × 10^6^	88.33	2.69 × 10^9^	8.95 × 10^7^	89%	3.02 × 10^9^	84%

**Table 3:** Comparison of hiPSC suspensions before and after concentration by kSep; v c, viable cell number.

	Unit	Run 1	Run 2	Run 3	Run 4	Run 5
**Pre-kSep VCD**	v c/mL	9.27 × 10^5^	1.86 × 10^6^	3.40 × 10^6^	3.30 × 10^6^	1.88 × 10^6^
**Pre-kSep Viability**	%	78.3	90.3	92.5	88.3	88.1
**Viable Cells into kSep**	v c	3.01 × 10^9^	6.07 × 10^9^	1.19 × 10^10^	9.12 × 10^9^	4.42 × 10^9^
**Volume Processed**	mL	3250	3265	3500	2760	2353
**Post-kSep VCD**	v c/mL	9.79 × 10^7^	7.88 × 10^7^	8.52 × 10^7^	2.26 × 10^8^	1.04 × 10^8^
**Post-kSep Viability**	%	86.6	89.9	91.3	93.0	86.8
**Viable Cells Harvested**	v c	2.94 × 10^9^	5.51 × 10^9^	1.08 × 10^10^	9.05 × 10^9^	4.14 × 10^9^
**Concentrated Volume**	mL	30	70	127	40	40
**Recovery**	%	97	91	91	99	94

**Table 4:** Cell viability of cryopreserved cells post thaw. Cells cryopreserved at various cell densities after harvest from a bioreactor and concentration by kSep were thawed, and cell viability was determined.

Vial #	Cell Line	Cells/Vial	Cell viability (%) before Cryopreservation	Cell Viability (%) Post Thaw
1	RTiPSC3B	4 × 10^6^	90	86
2	LiPSC18R	4 × 10^6^	87.3	78.2
3	LiPSC18R	40 × 10^6^	87.3	80.4
4	LiPSC18R	120 × 10^6^	87.3	79.4
5	LiPSC18R	240 × 10^6^	87.3	77.1

**Table 5:** Summary of cell expansion results obtained from 15 bioreactor runs.

Cell Line	Passage Number at Inoculation	Bioreactor Size	Inoculum Information	Inoculum Cell Density Cells/mL	Harvest Day	Expansion Fold
RTiPSC3B	P16	1L	Cells clumps harvested from 2D	0.04 × 10^6^	17	50
RTiPSC3B	P24	1L	Cells clumps harvested from 2D	0.04 × 10^6^	16	45
RTiPSC4i	P28	1L	Cells clumps harvested from 2D	0.04 × 10^6^	17	75
RTiPSC3B	P22	3L	Cells clumps harvested from 2D	0.04 × 10^6^	15	160
RTiPSC3B	P28	3L	Cells clumps harvested from 2D	0.04 × 10^6^	16	127
RTiPSC4i	P31	3L	Cells clumps harvested from 2D	0.04 × 10^6^	12	127
RTiPSC3B	P28	3L	Cells clumps harvested from 2D	0.04 × 10^6^	9	55
RTiPSC3B	P28	3L	Cells clumps harvested from 2D	0.04 × 10^6^	12	66
LiPSC18R	P26	3L	Cells clumps harvested from 2D	0.04 × 10^6^	9	64
LiPSC18R	P29	3L	Cells clumps harvested from 2D	0.04 × 10^6^	11	44
LiPSC18R	P34	3L	Cells clumps harvested from 2D	0.025 × 10^6^	12	112
RTiPSC3B	P29	3L	Cells clumps harvested from 2D	0.04 × 10^6^	15	82
RTiPSC4i	P30	3L	Cells clumps harvested from 2D	0.026 × 10^6^	11	72
LiPSC18R	P34	3L	Cryopreserved single cells	0.04 × 10^6^	17	47
LiPSC18R	P30	3L	Cryopreserved single cells	0.068 × 10^6^	9	50

## Abbreviations

hPSCs	Human pluripotent stem cells
hiPSCs	Human induced pluripotent stem cells
MCs	Microcarriers
EBs	Embryoid bodies
BSC	Bio Safety Cabinet
VVD	Vessel volume per day

## Appendix A

(A1) $$Concentrated\;Vol=\frac{\left(Feed\;VCD)*(Feed\;Vol\right)}{Target\;Concentrated\;VCD}$$
